# LncRNA RASAL2-AS1 promotes METTL14-mediated m6A methylation in the proliferation and progression of head and neck squamous cell carcinoma

**DOI:** 10.1186/s12935-024-03302-8

**Published:** 2024-03-25

**Authors:** Meiting Rong, Ming Zhang, Feihong Dong, Ke Wu, Bingkun Cai, Jinrui Niu, Le Yang, Zhongyan Li, Hui-yi Lu

**Affiliations:** 1 The Second Affiliated Hospital of Dalian Medical University, #467 Zhongshan Road, Dalian, 116023 China; 2 Tianjin Medical University Cancer Institute & Hospital, National Clinical Research Center for Cancer, Tianjin, 300060 China

**Keywords:** LncRNA RASAL2-AS1, METTL14, m6A methylation, Lis1, HNSCC

## Abstract

**Background:**

Long non-coding RNAs (lncRNAs) are key regulators of the 6-methyladenosine (m6A) epigenetic modification, playing a role in the initiation and progression of tumors. However, the regulatory mechanisms in head and neck squamous cell carcinoma (HNSCC) remain elusive. In this study, we investigated the molecular regulatory mechanisms of the lncRNA RASAL2-AS1 in the occurrence and development of HNSCC tumors.

**Methods:**

A bioinformatics analysis was conducted to analyze the expression level of RASAL2-AS1 in HNSCC and normal tissues. RASAL2-AS1 mRNA and protein levels were detected using RT-PCR and Western blotting. Wound healing, transwell assays, flow cytometry, M6A dot blot, and RNA immunoprecipitation experiments were conducted to explore the regulatory role of the RASAL2-AS1 and downstream targets METTL14/LIS1 signaling pathway in HNSCC. Immunohistochemical examination was conducted to evaluate the expression of METTL14 and LIS1 in HNSCC and normal tissues. A tumor xenograft model of BALB/c nude mice was established to assess the impact of RASAL2-AS1 on cell proliferation and growth.

**Results:**

RASAL2-AS1 high expression in HNSCC and cells deteriorated with survival rates of HNSCC. RASAL2-AS1 overexpression in HNSCC accelerated cell migration, colony formation, cell proliferation, cell cycle in S stage, while RASAL2-AS1 knockdown in HNSC cells inhibited cell cycle in G1 stage. After silencing METTL14, the above effects induced by overexpression of the RASAL2-AS1 were reversed. RASAL2-AS1 overexpression prompted LIS1 expression, whereas RASAL2-AS1 silencing reduced LIS1 levels in HNSCC cells, which was confirmed by immunohistological staining. Results demonstrated elevated expression of METTL14 or LIS1 in tongue cancer tissues. Overexpression of RASAL2-AS1 promoted tumor weight and tumor volume, which was counteracted by pcDNA3.1 RASAL2-AS1 plus silencing METTL14 and METTL14 and LIS1 were significantly decreased.

**Conclusion:**

Our study highlights the functional importance of the LncRNA RASAL2-AS1 in HNSCC and might assist in the development of a prognostic stratification and therapeutic approach. Which regulates HNSCC with the dependence of m6a manner.

**Supplementary Information:**

The online version contains supplementary material available at 10.1186/s12935-024-03302-8.

## Introduction

Head and neck squamous cell carcinoma (HNSCC) has a complex pathogenesis, characterized by a high rate of recurrence and distant metastasis [1] is ranked as the sixth most prevalent cancer worldwide [2]. Oral squamous cell carcinoma (OSCC) is the most common subtype of HNSCC, with approximately 350,000 new cases and 170,000 deaths worldwide in 2018 [3]. Cisplatin-based CCRT is still considered to be the first-line treatment for locally advanced HNSCC [4]. Immune regulation plays a pivotal role in the development and progression of tumors [5–9]. Consequently, nivolumab and pembrolizumab are also approved for treatment in the platinum-failure setting for R/M HNSCC patients [10]. Low-Dose Hsp90 Inhibitor Selectively Radiosensitizes HNSCC and Pancreatic Xenografts [11]. Although technological advances in surgery, radiotherapy and chemotherapy have improved local control of HNSCC, the 5 year survival rate remains low [12]. The new therapies for tumor treatment such as immunotherapy techniques developed in recent years have brought blessings to HNSCC patients, they have also brought serious side effects and financial burdens to patients [13]. Therefore, it is crucial to find a new breakthrough in the treatment of HNSCC.

Long non-coding RNAs (lncRNAs) represent a novel class of noncoding RNA molecules, often defined as being more than 200 nucleotides in length [14]. LncRNAs have attracted much scientific attention due to their high abundance but limited protein-coding capacity. Numerous studies have demonstrated that lncRNAs are involved in many biological processes of tumor, including immune response, proliferation and metastasis of tumor cells [15]. RASAL2-AS1 (RASAL2 antisense RNA 1), located in chromosome 1q25.3, is a natural antisense lncRNA with 2485nt length. RASAL2-AS1 was revealed a methylation-driven gene associated with the occurrence and progression of hepatocellular carcinoma, basing on cancer genomics profiles by Li J et al [13]. Afterwards, a joint analysis of lncRNA m6A methylome and lncRNA/mRNA expression profiles was conducted in gastric cancer, revealing that RASAL2-AS1 is an m6A methylation-related lncRNA [14]. However, the role of RASAL2-AS1 in the HNSCC has not been clearly investigated.

Current interest is increasingly directed towards reversible post-transcriptional modifications of RNA. Among these, the N6‐methyadenosine (m6A) modification is the most abundant and conserved type of mammalian mRNA methylation [15] that is involved in the regulation of gene expression by modulating RNA processing, localisation, translation and eventual decay [16, 17]. M6A modification is mainly regulated by methyltransferases (METTL3, METTL14 and RBM15), demethylases (ALKBH5 and FTO) and RNA reading proteins (EIF3, YTHDC1 and HNRNPC) [18]. Methyltransferase-like 14 (METTL14) is the central component of the m6A methylated transferase complex, which is involved in the dynamic reversible process of m6A modification. METTL14 acts as both an oncogene and tumor suppressor gene to regulate the occurrence and development of various cancers. The abnormal m6A level induced by METTL14 is related to tumorigenesis, proliferation, metastasis, and invasion [19].

Tumorigenesis is orchestrated through complex interactions among multiple signaling pathways, each of which plays a pivotal role in regulating cell growth, differentiation, and apoptosis. These pathways, when dysregulated, contribute to the uncontrolled cellular proliferation and resistance to cell death [19–28]. LIS1 is a microtubule-organizing center-associated protein that regulates the polymerization and stability of microtubules by mediating the motor function of dynein. Recent studies have suggested that LIS1 plays a potential role in the malignant development of tumors, such as in mitosis and migration [20–23]. However, whether the lncRNA RASAL2-AS1 is involved in the regulation of HNSCC occurrence and development through interaction with METTL14, targeting downstream LIS1 mRNA in an m6A-dependent manner, remains to be elucidated.

Herein, this study demonstrated that LncRNA RASAL2-AS1 was notably highly expressed in both Cal-27 and SCC-25 cells and HNSCC, and augmented RASAL2-AS1 was proven to accelerate HNSCC progression and benefit for clinical diagnosis. The biological relationship between RASAL2-AS1 and HNSCC survival was certified using bioinformatics methods. In addition, METTL14 was verified to be a crucial m6A methyltransferase to preserve the stability of RASAL2-AS1. Our study revealed that RASAL2-AS1 overexpression contributes to the carcinogenic process by enhancing cell proliferation, migration, and colony formation in HNSCC. Silencing METTL14 reversed the effects induced by RASAL2-AS1 overexpression. RASAL2-AS1 overexpression prompted LIS1 expression. LIS1 was downstream target of RASAL2-AS1/ METTL14, RASAL2-AS1 silencing reduced LIS1 levels in HNSCC cells. METTL14 or LIS1 in tongue cancer tissues. Upregulated RASAL2-AS1 promoted tumor weight and tumor volume, which was counteracted by silencing METTL14. These aforementioned studies suggest that RASAL2-AS1 provides a promising target for the HNSCC prognostics and treatment.

## Method

### Bioinformatics analysis

Initial data regarding RASAL2-AS1 were obtained from the UCSC XENA database (https://xena.ucsc.edu/), which provided a rich repository of genomic and transcriptomic information. Subsequently, whole-genome expression profiles along with clinicopathological data of cancer patients were downloaded from The Cancer Genome Atlas (TCGA) (https://tcga-data.nci.nih.gov/). The lncRNAs were identified by the Gencode database (https://www.gencodegenes.org/). Data processing and analysis were conducted using the R programming language (https://www.r-project.org/, version 4.1.1), leveraging the ‘tidyverse’ package for data manipulation and the ‘DESeq2’ package for differential expression analysis. To explore the association between RASAL2-AS1 expression, patient survival in head and neck squamous cell carcinoma (HNSC), and potential downstream targets, the GEPIA database (http://gepia.cancer-pku.cn/) was utilized.

### Cell culture and cell treatment

Cal27, SCC-25, and SCC-15 cell lines, sourced from the American Type Culture Collection (ATCC, Manassas, VA), were utilized in our study. Cal27 cells were cultivated in Dulbecco’s Modified Eagle’s Medium (DMEM, ATCC), while SCC-15 and SCC-25 cells were maintained in MEM NEAA medium. All cell cultures were incubated at 37 °C in a 5% CO2 atmosphere. Antibiotics used included a penicillin-streptomycin-gentamicin mix (100×, P1410-100 ml) and a penicillin-streptomycin solution (100×, P1400-100 ml) to prevent contamination.

### Plasmid construction and cell transfection

This article was conducted under normal oxygen conditions.In this article, pcDNA3.1 empty plasmid was used as the control group, while pcDNA3.1 RASAL2-AS1 overexpressing RASAL2-AS1 was used as the experimental group.The RASAL2-AS1 plasmid was constructed and synthesized from the Jima gene, with a gene length of 2456 bp.The cloning vector was pcDNA3.1 (+) (GenePharma, Suzhou, China) and the cloning site was BamHI EcoRI. Cell transfection: Cal-27 cells and SCC-25 cells were evenly spread in a six-well plate at 3.0 × 105/mL, so that the cell density could reach 70% during transfection. Dilute 5µL Lipo2000 (Invitrogen, USA) or Lipofiter (Hanhen Biologicals) with 250µL Opti-MEM I Reduced Serum Medium, mix well, and let stand at room temperature for 5 min. Dilute 8µL si-RASAL2-AS1 or pcDNA3.1 RASAL2-AS1 and si-NC or pcDNA3.1 with 250µL Opti-MEM I Reduced Serum Medium, respectively, and let stand at room temperature for 5 min. The prepared Lipo2000 or Lipofiter was mixed with si-RNA or plasmid and left for 20 min at room temperature. Add 1 mL PBS to each well to clean twice, suck out PBS, and add 1.5 mL indual antibody medium. Then the prepared mixture of Lipo2000 or Lipofiter and si-RNA or plasmid 500µL was added into 6-well plates according to the groups, mixed well, and cultured in CO2 incubator at 37℃ for 6 h. After 6 h, the culture medium was changed into complete medium containing 10% serum and continued to be cultured for 48 h. Protein extraction, RNA extraction and subsequent phenotypic experiments were performed.

### Methyl thiazolyl tetrazolium (MTT) proliferation assay

Stably transfected cells were plated in 96-well plates at equal numbers. Cells were seeded at a density of 4.0 × 10^4^ cells per milliliter. The negative control group consisted of untransfected cells, while the experimental group comprised transfected cells. Equal amount of PBS buffered salt solution was added to the edge wells. 50 µL MTT solution (Elabscience Biotechnology, Wuhan, China) was added into cells of each well. The plates were placed into an incubator with 5% CO2 at 37 °C for 4 h. After removing the culture medium, the precipitate was dissolved in 150 µL of DMSO. The absorbance at 570 nm was measured to evaluate cell viability using the SpectraMax 190 Microplate Reader (Anthos Labtec Instruments, Austria).

### Cellular RNA extraction and RT-qPCR

Total RNA was extracted by Ezol (GenePharma, Suzhou, China). The concentration and purity of RNA were detected using a NANODROP-type quantitative nucleic acid analyzer. mRNA was reverse transcribed into cDNA using the TransGen Biotech (Beijing, China) and Accurate Biotechnology (Hunan, China). A 20 µL reaction system was prepared to facilitate cDNA synthesis.

Reverse transcription-polymerase chain reaction (RT-PCR) analysis was conducted following the guidelines provided by TransGen Biotech (Beijing, China) and Accurate Biotechnology (Hunan, China), utilizing PerfectStart Green qPCR SuperMix and SYBR Green Pro Taq HS premixed qPCR kit instructions. The internal reference for detection was GAPDH, and each sample was subjected to three repetitions. Subsequently, the Ct value was recorded, and the expression of the target genes was computed. Relative expressions were determined using the 2 ^−ΔΔCt^ method. The primer sequences for LncRNA RASAL2-AS1, METTL14, LIS1, and GAPDH are elaborated in Table S1.

### Cell migration, invasion and wound healing assays

Transwell chambers (8 mm pore size; Corning Costar, USA) were used to measure cell migration ability according to the vendor’s instructions. Cal-27 and SCC-25 cells (10^5^ cells/well) were seeded into the upper chambers, reaching 70–80% confluence cell transfections were carried out. After 48 h, the cells were fixed with 4% paraformaldehyde for 30 min and stained with 0.1% crystal violet for 30 min. The migrated cells in the upper chambers were photographed under a phase-contrast microscope and counted.

In wound healing assay, cells were seeded at a density of 1 × 10^6^ cell/well onto six-well plates and cultured to about 80–90% confluence, and then a sterile 200 µl pipette tip a scratch was used to form artificial scratches for each well. The suspended cells were washed away with PBS, and then the cells were cultured in medium with 1% FBS. Images of a specific position on the scratched areas were taken using an inverted microscope (Leica, GER) at 0 h, 24 h, and 48 h.

### Flow cytometry analysis

Transfected Cal-27 and SCC-25 cells were seeded at the density of 5 × 10^5^ cells per well. Then cells were placed in 6-well plates using cell cycle assay kit from keyGEN BioTECH (Nanjing, China). F According to the instructions, prepare the RnaseA:PI working solution in a 1:9 volume ratio to create the staining working solution, using 500 µL for each sample. First, the cells were washed once with PBS, then the cells were centrifuged at 2000 rpm for 5 minutes. Afterward, 1 ml of single-cell suspension was collected and centrifuged to prepare suspension and remove the supernatant. Add 70% cold ethanol to the preconfigured PI/RNaseA staining working solution, take 500 µL of this mixture and add it to the cell suspension. Prior to staining, filter the solution once through a 300-mesh sieve. Finally, utilize a self-service flow cytometer (Agilent, UAS) to analyze the samples.

### Western blot assay

Cultured cells were lysed in RIPA lysis buffer, and the collected total proteins were separated using 10% SDS-PAGE and transferred onto a PVDF membrane. After blocking with 5% skim milk, the primary antibodies for METTL14 CST (1:500 dilution; Rabbit mAb 26158-1-AP) and LIS1 ABclonal (1:500 dilution; Rabbit mAb A3696) were incubated overnight at 4 °C. Secondary antibodies conjugated with H + L were obtained from Invitrogen and incubated with the membrane at room temperature for 2 h. After washing three times with TBST, the ECL detection system (BIO-RAD, USA) was used following the user manual for detection.

### Dot blot assay

A micropipette was used to apply protein droplets onto the PVDF membrane and dried with air. The membrane was blocked with 5% BSA in TBST solution for 1 h, and then incubated with primary antibodies against METTL14 CST (1:500 dilution; Rabbit mAb 26158-1-AP) and LIS1 ABclonal (1:500 dilution; Rabbit mAb A3696) at room temperature for 30 min. Then the membrane was washed three times with 1% TBST, after incubated with secondary antibodies H + L at room temperature for 30 min, followed by three times with 1% TBST with the user manual (BIO-RAD, USA).

### Agarose gel electrophoresis

Weigh agarose and dissolve it in 1X TAE Buffer. Heat the solution in a microwave until it reaches 50 °C. Add 5 µL of nucleic acid dye and mix well. Pour the mixture into a gel casting tray and let it solidify. Place the solidified gel into an electrophoresis chamber and cover it with TAE buffer. Load the samples onto the gel and start the electrophoresis process. After electrophoresis, use a gel imaging system (BIO-RAD, USA) to capture images.

### RNA immunoprecipitation (RIP) analysis

RIP (RNA Immunoprecipitation) assays were conducted using the Jiesalin RIP Kit from Guangzhou Jiesalin Biotechnology Co., Ltd.,. The cell lysate was incubated with magnetic beads bound to IgG antibodies. Immunoprecipitated RNA was validated through RT-qPCR and gel electrophoresis. Input RNA was used as a positive control.

### Tumor xenograft assay

Nude BALB/c mice (female, 4 weeks old) were purchased from Vital River Laboratories (VRL) (Beijing, China) and raised under specific pathogen-free (SPF) conditions. Mice were randomly divided into 3 groups (*n* = 6 for each group) and subcutaneously injected with SCC-25 cells by stable co-transfection with pcDNA3.1, pcDNA3.1 RASAL2-AS1, and pcDNA3.1 RASAL2-AS1 + si-METTL14. Starting from the first injection, tumor volume was measured every 4 days for 28 days. Tumor tissues were harvested for hematoxylin and eosin (H&E) and immunohistochemistry (IHC) staining. Female mice were used in the experiment mainly because the experiment period was long, the temperament of female mice was mild, and the male mice were easy to fight together, which would cause injury and death to affect the experimental results. Therefore, female nude mice were used in the experiment. All animal experimental procedures adhered to institutional ethical requirements and were approved by the Dalian Medical University Animal Use and Care Committee.The endpoints for in vivo experiments are defined as follows: the termination of animal life is warranted when an animal’s body weight decreases by 20–25%, or if the animal exhibits cachexia or wasting syndrome, or when the size of a solid tumor exceeds 10% of the animal’s body weight.

### Immunohistochemistry analysis (IHC)

#### Patient samples

Twelve tongue cancer tissue and five specimens of normal tissue were harvested from the second Affiliated with Dalian medical University School of Medicine. The patient studies were conducted in accordance with Declaration of Helsinki. The specimens and data for research purposes were granted approval by the Ethics Committee of the second Affiliated Hospital of Dalian Medical University (Expedited review by The Second Affiliated Hospital of Dalian Medical University in 2023 No.180).

Tissue samples from patients with head and neck squamous cell carcinoma and mouse models were collected, fixed, and embedded in paraffin. Subsequently, 4 μm sections were deparaffinized and rehydrated. Immunohistochemical staining was performed using anti-METTL14 CST (1:50 dilution; Rabbit mAb 26158-1-AP) and anti-LIS1 antibodies ABclonal (1:50 dilution; Rabbit mAb A3696). The research protocol involving patient samples was approved by the Ethics Committee of the Second Hospital of Dalian Medical University.

### Data analysis

This study employed GraphPad Prism software v 9.3 (GraphPad Software, San Diego, CA, USA) for statistical analysis. All calculations are presented as mean ± standard deviation (SD). Differences between two groups or more were analyzed using Student’s t-test or one-way/two-way ANOVA. A p-value < 0.05 was considered statistically significant. All experiments were performed in triplicate.

## Result

### Correlation of LncRNA RASAL2-AS1 with m6A methylated METTL14 expression in HNSCC tissues and cells

**Fig. 1:**
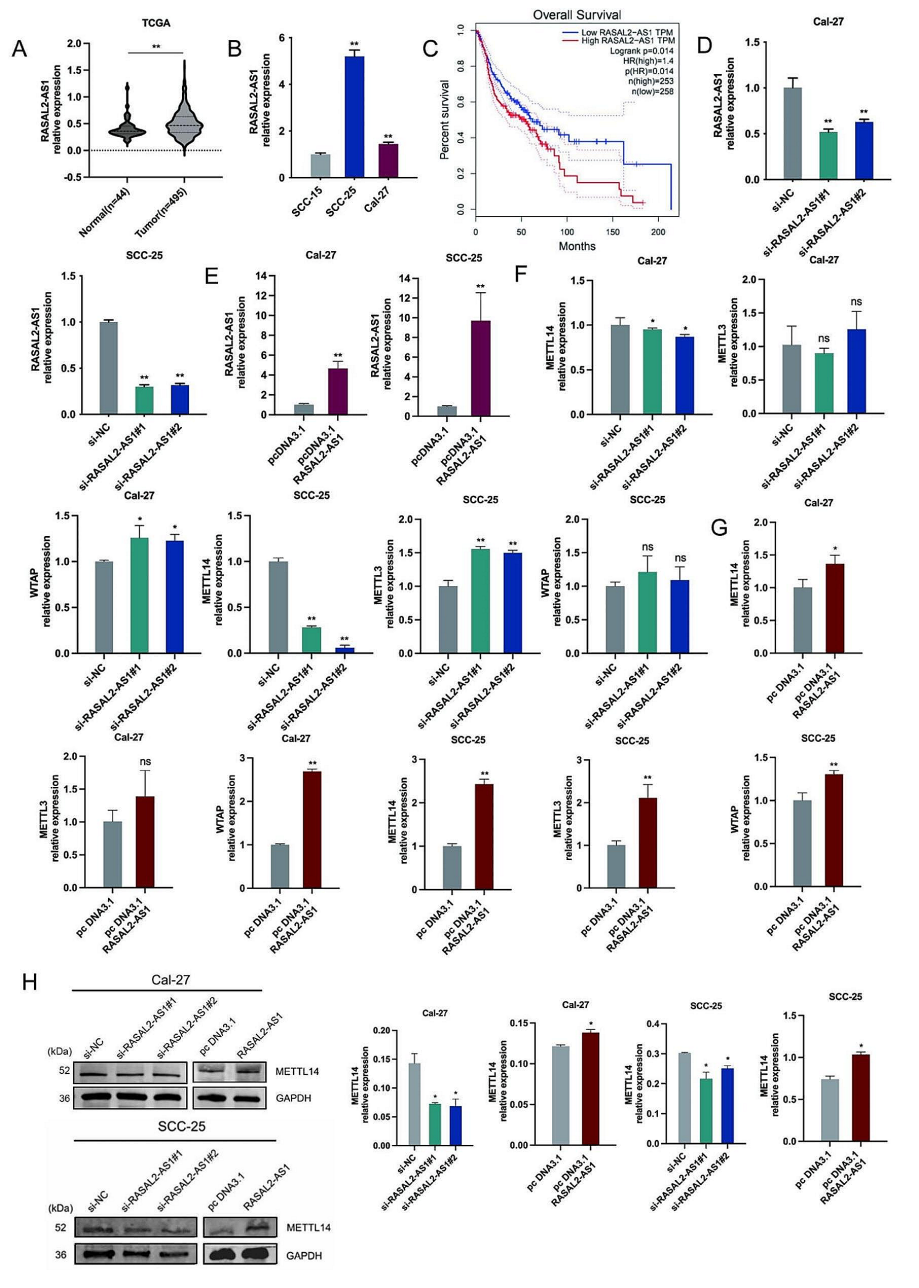
Correlation of LncRNA RASAL2-AS1, m6A, METTL14 expression in HNSC tissues and cells. (**A**) Expression of RASAL2-AS1 in 495 HNSC tissues and 44 normal tissue samples. (**B**) Expressions of RASAL2-AS1 mRNA in HNSCC cells. (**C**) Predicted survival curves of RASAL2-AS1 in relation to patient survival. (**D**-**E**) Silencing and overexpression efficiency of RASAL2-AS1 mRNA in cells. (**F**-**G**) Levels of METTL14, METTL3, WTAP mRNA in cells after silencing and overexpression of RASAL2-AS1. (**H**) The protein levels of METTL14 in cells after silencing and overexpression of RASAL2-AS1. ^*^p < 0.05, ^**^p < 0.01

To investigate the role of RASAL2-AS1 in the pathogenesis of HNSCC, we analyzed its expression in 495 HNSCC and 44 non-HNSCC tissue samples using the UCSC XENA database. This analysis revealed that RASAL2-AS1 levels were significantly higher in HNSCC tissues than in normal ones (Fig. 1A). RT-PCR comparisons showed pronounced overexpression of RASAL2-AS1 in Cal-27 and SCC-25 cells relative to SCC-15 cells (Fig. 1B), leading us to focus further on these cell lines. We then used the GEPIA tool to assess the correlation between RASAL2-AS1 expression and patient survival, finding that higher RASAL2-AS1 levels were linked to poorer overall survival in HNSCC patients (Fig. 1C). To experimentally alter RASAL2-AS1 expression, we conducted transient knockdown and overexpression in Cal-27 and SCC-25 cells, with RT-qPCR confirming effective downregulation in si-RASAL2-AS1 groups compared to the si-NC control group (Fig. 1D). Conversely, pcDNA3.1RASAL2-AS1 significantly upregulated RASAL2-AS1 compared to pcDNA3.1, demonstrating successful overexpression (Fig. 1E). HNSCC clinical samples categorized based on RASAL2-AS1 expression showed correlations with patient survival and clinical stage N (Table S2); however, no significant associations were found with gender, clinical stage M, T stage, clinical stage, or tumor grade. In our investigation of the interaction between RASAL2-AS1 and m6A-methylated METTL14 expression, RT-qPCR revealed reduced METTL14 expression upon RASAL2-AS1 silencing and increased expression upon overexpression (Fig. 1F and G). Western blot assays illustrated the impact of RASAL2-AS1 modulation on METTL14 protein expression (Fig. 1H). These findings provide valuable insights into the role of RASAL2-AS1 in HNSCC pathogenesis, its association with patient survival, and its interaction with METTL14, a gene involved in m6A methylation.

### Impact of RASAL2-AS1 on HNSCC Cell characteristics

**Fig. 2:**
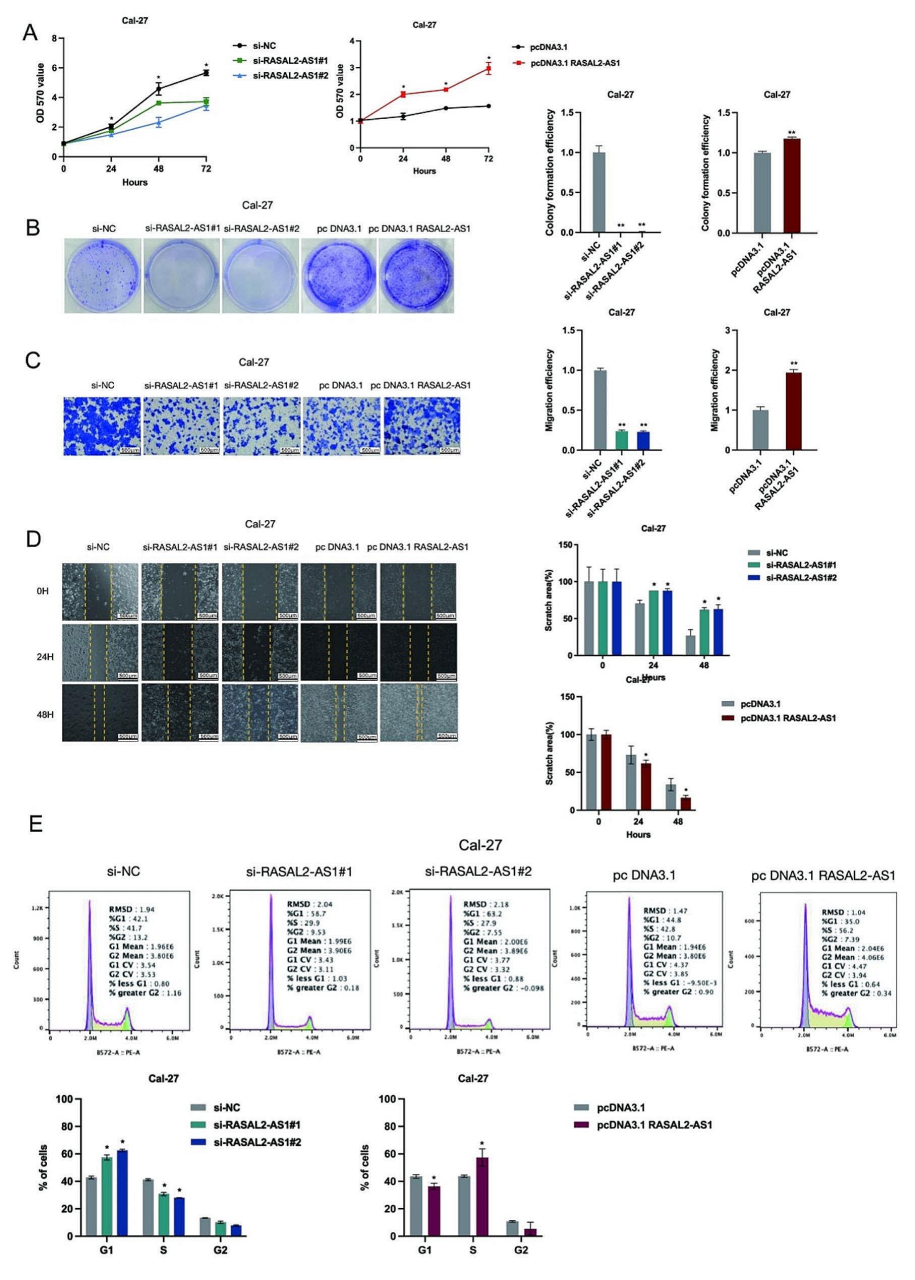
Effect of RASAL2-AS1 on HNSCC cell proliferation, colony formation, migration and cycle. (**A**) Cell viability of RASAL2-AS1 after silencing or overexpression in Cal-27 and SCC-25 cells. (**B**) The colony formation ability of Cal-27 and SCC-25 cells after silencing or overexpression of RASAL2-AS1. (**C**-**D**) The migration ability of Cal-27 and SCC-25 cells after silencing or overexpression of migration ability of Cal-27 and SCC-25 cells after silencing or overexpression of RASAL2-AS1 (scale bars, 500 μm). (**E**) The cycle of Cal-27 and SCC-25 cells after silencing or overexpression of RASAL2-AS1. ^*^p < 0.05, ^**^p < 0.01

Our study conducted a range of cell phenotyping experiments to ascertain the impact of RASAL2-AS1 on HNSCC cell traits. The MTT assay revealed that RASAL2-AS1 downregulation significantly curbed the proliferation of Cal-27 and SCC-25 cells. In contrast, enhanced RASAL2-AS1 expression notably boosted their proliferation at 24, 48, and 72 h (Fig. 2A). Colony formation assays further indicated that RASAL2-AS1 knockdown notably reduced colony-forming abilities in these cells, whereas its upregulation significantly fostered proliferation (Fig. 2B).

Additionally, transwell and scratch assays showed that inhibiting RASAL2-AS1 greatly decreased the migration of Cal-27 and SCC-25 cells, while its overexpression markedly increased this ability (Fig. 2C, D). Flow cytometry analysis for cell cycle dynamics demonstrated that RASAL2-AS1 silencing predominantly arrested the cell cycle in the G1 phase, a process reversed by its overexpression (Fig. 2E). These findings collectively underscore the considerable influence of RASAL2-AS1 on various biological aspects of HNSCC cells.

### RASAL2-AS1 and METTL14 interactions in HNSCC cells

**Fig. 3:**
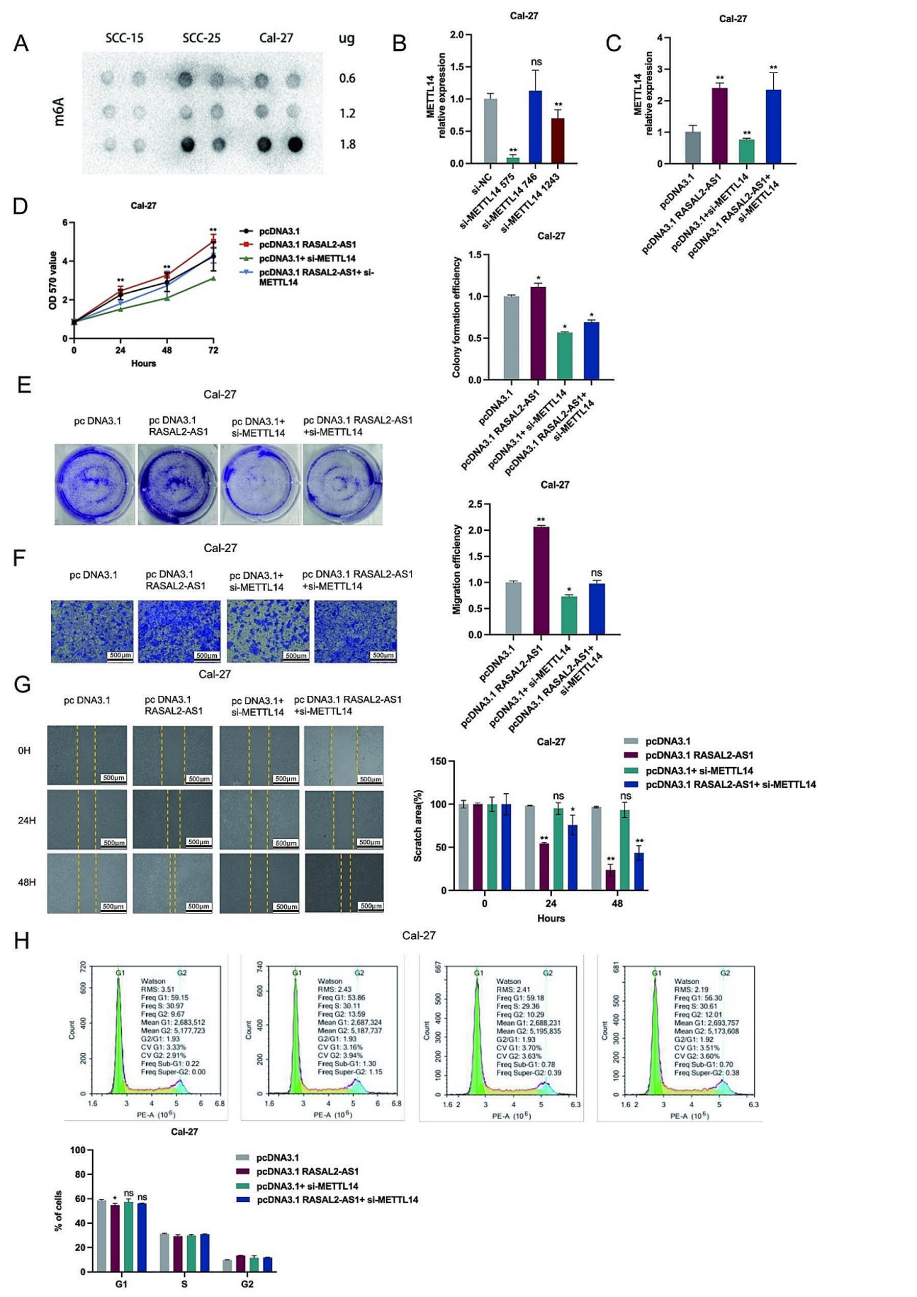
Overexpression of RASAL2-AS1 restored the proliferation inhibition of HNSCC cells induced by silencing METTL14. (**A**) Levels of m6A in HNSCC cells (including SCC-15, SCC-25 and Cal-27) were detected by m6A Dot blot. (**B**) The optimal sequence of METTL14 silencing efficiency. The expressions of METTL14 mRNA (**C**), (**D**) the cell viability, (**E**) colony formation ability, (**F**) migration ability, (**G**) scratch area and (**H**) cell cycle after co-transfected overexpression vector RASAL2-AS1 and silenced METTL14 in Cal-27 and SCC-25 (scale bars, 500 μm). ^*^p < 0.05, ^**^p < 0.01

To understand the regulatory effects and interactions of RASAL2-AS1 on METTL14 and its potential biological functions in HNSCC cells, we conducted rescue experiments by suppressing METTL14 in RASAL2-AS1 overexpressing HNSCC cells. Initially, M6A dot blot assays indicated higher levels of m6A methylation in SCC-25 and Cal-27 cells compared to SCC-15 cells (Fig. 2A). The effectiveness of METTL14 silencing was confirmed through RT-qPCR (Fig. 3B). Subsequently, a restoration of expression was observed in Cal-27 and SCC-25 cells following co-transfection with the RASAL2-AS1 overexpression vector and the METTL14 silencing vector (Fig. 3C). In vitro recovery assays consistently demonstrated that the proliferative capacity, colony formation ability, migratory potential, and cell cycle effects of Cal-27 and SCC-25 HNSCC cells were restored to baseline levels through MTT (Fig. 3D), colony formation assays (Fig. 3E), transwell assays (Fig. 3F), scratch assays (Fig. 3G), and flow cytometry (Fig. 3H). Silencing METTL14 expression reversed the enhanced biological properties in HNSCC resulting from RASAL2-AS1 overexpression. Therefore, these findings indicated that suppressing METTL14 expression can effectively counteract the biological effects induced by overexpression of the RASAL2-AS1 gene. These sets of experiments elucidates the regulatory role of RASAL2-AS1 on METTL14 and how it influences various biological characteristics in HNSCC cells. It also demonstrates that targeting METTL14 can reverse the effects of RASAL2-AS1 overexpression, providing valuable insights into potential therapeutic strategies for HNSCC.

### LIS1 is critical downstream targets of METTL14 regulated by RASAL2-AS1 in HNSCC cells

**Fig. 4:**
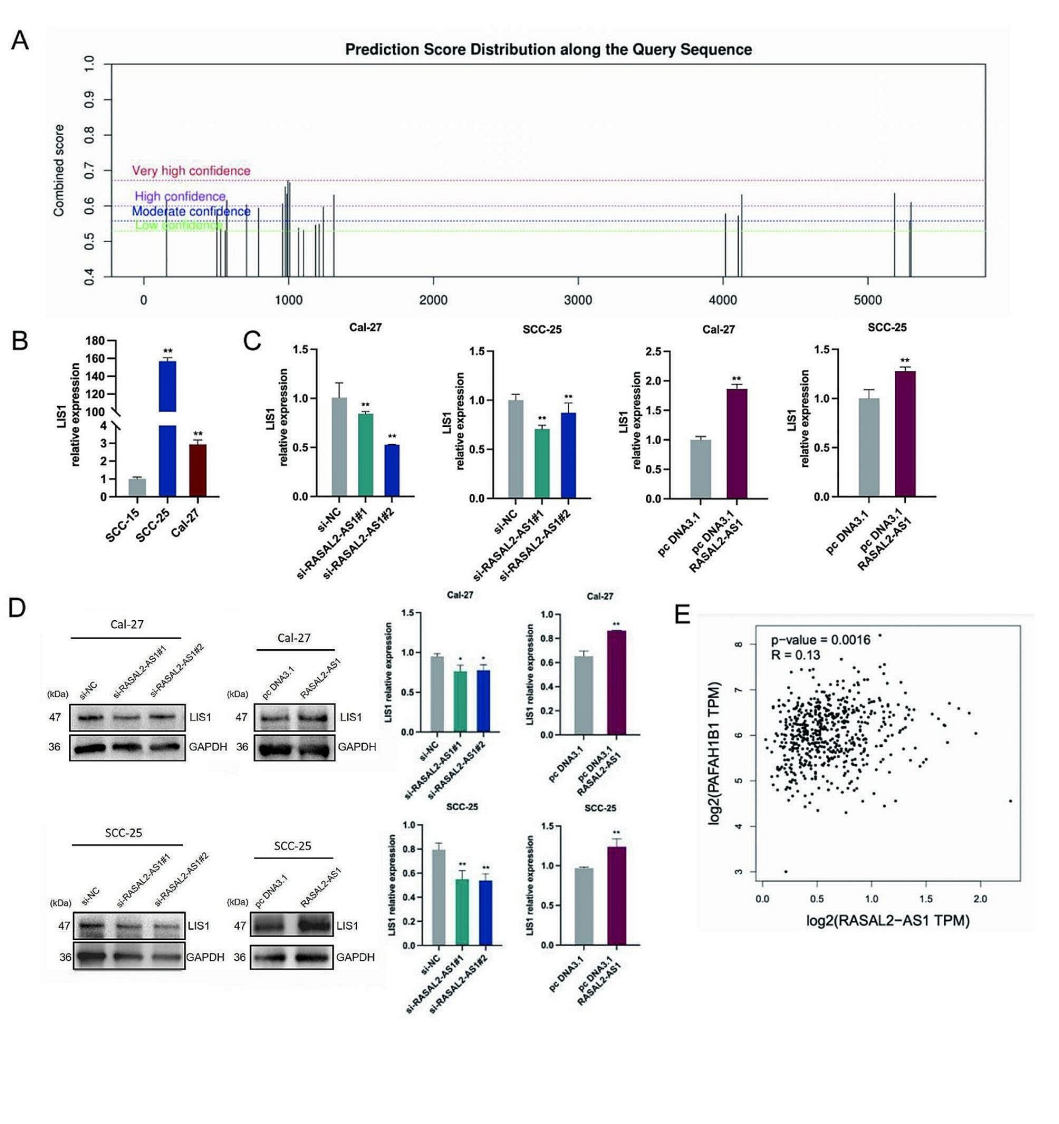
Silencing of RASAL2-AS1 suppresses RNA stability and expression of LIS1. (**A**) SRAMP predicts the m6A site on the secondary structure of LIS1 mRNA. (**B**) Levels of LIS1 mRNA in SCC-15, SCC-25, and Cal-27 cells. (**C**-**D**) LIS1 mRNA and protein levels after silencing or overexpression of RASAL2-AS1 in Cal-27 and SCC-25 cells. (**E**) A positive correlation between LIS1 expression and RASAL2-AS1 expression. ^*^p < 0.05, ^**^p < 0.01

To investigate downstream genes influenced by METTL14-mediated m6A methylation in HNSCC progression under RASAL2-AS1 regulation, we employed SRAMP to predict LIS1’s interaction with m6A methylation sites. This analysis identified m6A methylation sites with high confidence levels (Fig. 4A). Subsequent RT-qPCR analysis revealed LIS1 expression in both HNSCC cell types, notably elevated in Cal-27 and SCC-25 cells (Fig. 4B). LIS1 emerged as a promising target candidate. Silencing RASAL2-AS1 in Cal-27 and SCC-25 cells resulted in significant downregulation of LIS1, while RASAL2-AS1 overexpression led to substantial upregulation of LIS1 (Fig. 4C). These findings were further corroborated by western blot analysis, which demonstrated a positive regulatory pattern of LIS1 protein levels by the modulation of RASAL2-AS1 expression (Fig. 4D). Specifically, RASAL2-AS1 overexpression increased LIS1 protein expression, whereas RASAL2-AS1 silencing temporarily reduced LIS1 protein levels in HNSCC cells. Additionally, the GEPIA platform hinted at a positive correlation between RASAL2-AS1 and LIS1 (PAFAH1B1) (Fig. 4E). These findings highlight LIS1 as a potential novel direct target of METTL14-mediated m6A methylation, orchestrated by RASAL2-AS1 in the context of HNSCC progression.

#### RASAL2-AS1-METTL14-LIS1 signaling axis in HNSCC cells and tissues

**Fig. 5:**
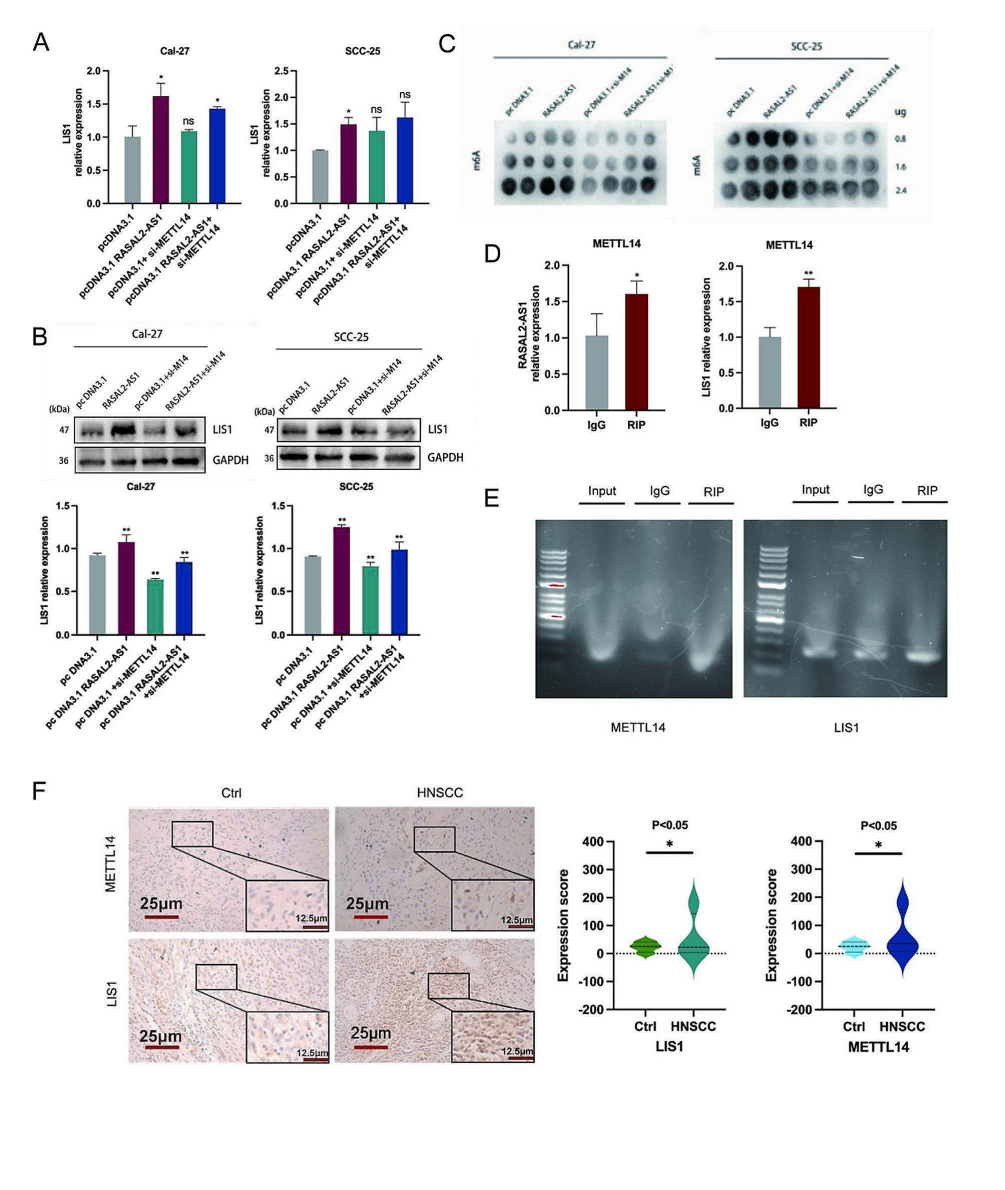
RASAL2-AS1 overexpression restored the LIS1 expression inhibition induced by METTL14 silencing. (**A**-**B**) LIS1 mRNA levels and protein expression in Cal-27 and SCC-25 cells cotransfected with the overexpressed RASAL2-AS1 vector and silenced METTL14 levels. (**C**) The level of m6A methylation modification by overexpression of RASAL2-AS1 vector and silencing of METTL14 in Cal-27 and SCC-25 cells. (**D**-**E**) The triple RASAL2-AS1-METTL14-LIS1 binding. (**F**) Representative images of IHC staining of the expression of METTL14 and LIS1 in HNSCC tissues are shown (Original magnification ×200 scale bars, 25 μm; ×400 scale bars,12.5 μm). ^*^p < 0.05, ^**^p < 0.01

To delve deeper into the critical role of LIS1 regulation by METTL14 and RASAL2-AS1, we conducted recovery experiments. These experiments revealed that the mRNA and protein expression of LIS1 was restored in the presence of RASAL2-AS1 overexpression and METTL14 silencing (Fig. 5A-B). Furthermore, m6A dot blot analysis indicated that, depending on the concentration of protein (0.8, 1.6, and 2.4 µg), higher m6A methylation levels were detected in the RASAL2-AS1 overexpression group, and lower levels were detected in the METTL14 silencing group compared to the pcDNA3.1 group. However, in the co-transfection of overexpression RASAL2-AS1 and silencing METTL14, m6A methylation levels were restored (Fig. 5C). Additionally, results from RNA immunoprecipitation (RIP) and agarose gel electrophoresis indicated a binding relationship between RASAL2-AS1, METTL14, and LIS1 (Fig. 5D-E). Immunohistochemistry experiment findings revealed elevated expression of METTL14 and LIS1 in tongue cancer patients compared to normal tissues (Fig. 5F). These results support the existence of a novel key signaling axis, RASAL2-AS1-METTL14-LIS1, which plays a regulatory role in m6A methylation modification levels and RNA stability during the development of HNSCC. This axis appears to be crucial in understanding the underlying mechanisms of HNSCC progression.

#### RASAL2-AS1 promoted cells proliferation and growth by targeting METTL14 in vivo

**Fig. 6:**
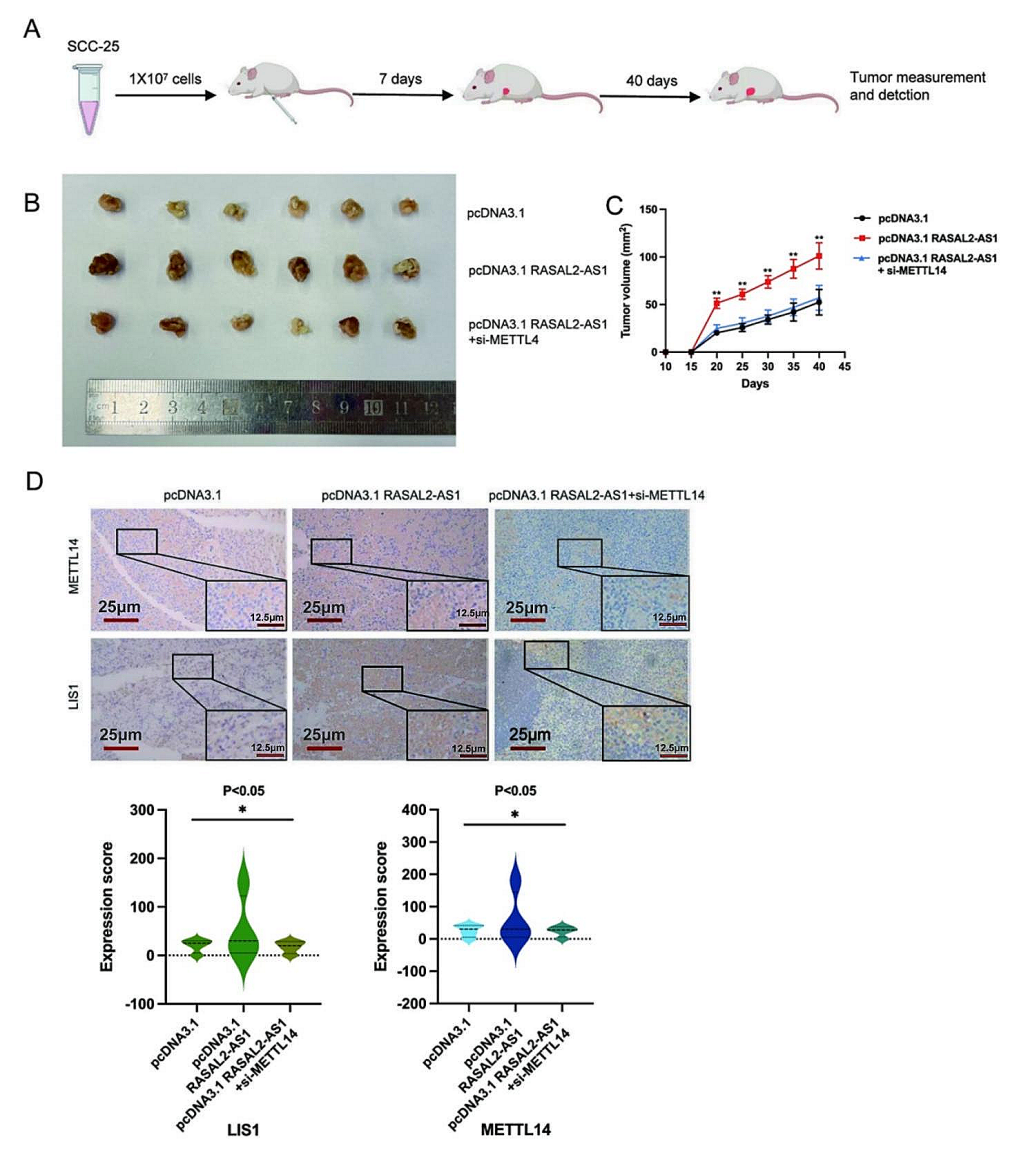
RASAL2-AS1 promotes HNSCC cell proliferation and growth by targeting METTL14 in vivo. (**A**) Xenograft plots in BALB/c mice by inoculating the right axilla with pcDNA3.1, pcDNA3.1 RASAL2-AS1, and pcDNA3.1 RASAL2-AS1 + si-METTL14 cotransfected SCC-25 cells, respectively. (**B**) Plots show xenograft tumors excised from mice on day 40. (**C**) Determination of each group of tumor volumes of nude mouse xenograft tumors at different days (*n* = 6 for each group). (**D**) Representative images of IHC staining of the expression of METTL14 and LIS1 in HNSCC tissues are shown (Original magnification ×200 scale bars, 25 μm; ×400 scale bars, 12.5 μm). ^*^p < 0.05, ^**^p < 0.01

To assess the molecular mechanism of RASAL2-AS1-METTL14 interaction in HNSCC in vivo, we conducted experiments using SCC-25 cells that were stably co-transfected with pcDNA3.1, pcDNA3.1 RASAL2-AS1, and pcDNA3.1 RASAL2-AS1 + si-METTL14 in BALB/c nude mice to establish a tumor xenograft model (Fig. 6A). In vitro assessment of xenograft tumor weight and tumor volume revealed significant differences between the treated mice and the control group after 5–6 weeks of growth (Fig. 6B). Additionally, xenograft tumor volumes were monitored every 4 days, and the pcDNA3.1 RASAL2-AS1 group exhibited the fastest growth rate compared to the control group. However, there was no significant difference in tumor volumes after treatment with the pcDNA3.1 RASAL2-AS1 + si-METTL14 vector compared to the control group (Fig. 6C). To further confirm these findings, immunohistochemical (IHC) analysis showed that METTL14 was significantly increased in the pcDNA3.1 RASAL2-AS1 group. Notably, the expression of the target gene LIS1 was similarly significantly elevated in the pcDNA3.1 RASAL2-AS1 group. However, METTL14 and LIS1 were significantly decreased in the pcDNA3.1 RASAL2-AS1 + si-METTL14 group (Fig. 6D). This suggests that the promoting effect on tumor growth is a result of the interaction of RASAL2-AS1 with METTL14 in the xenograft model. Thus, in vivo studies demonstrated that the overexpression of RASAL2-AS1 promoted HNSCC cell proliferation and growth, while silencing METTL14 inhibited HNSCC cell proliferation and growth. These findings provide that RASAL2-AS1 targets METTL14 to regulate the LIS1 signaling pathway in vivo, offering valuable insights into the role of this interaction in HNSCC progression.

**Fig. 7:**
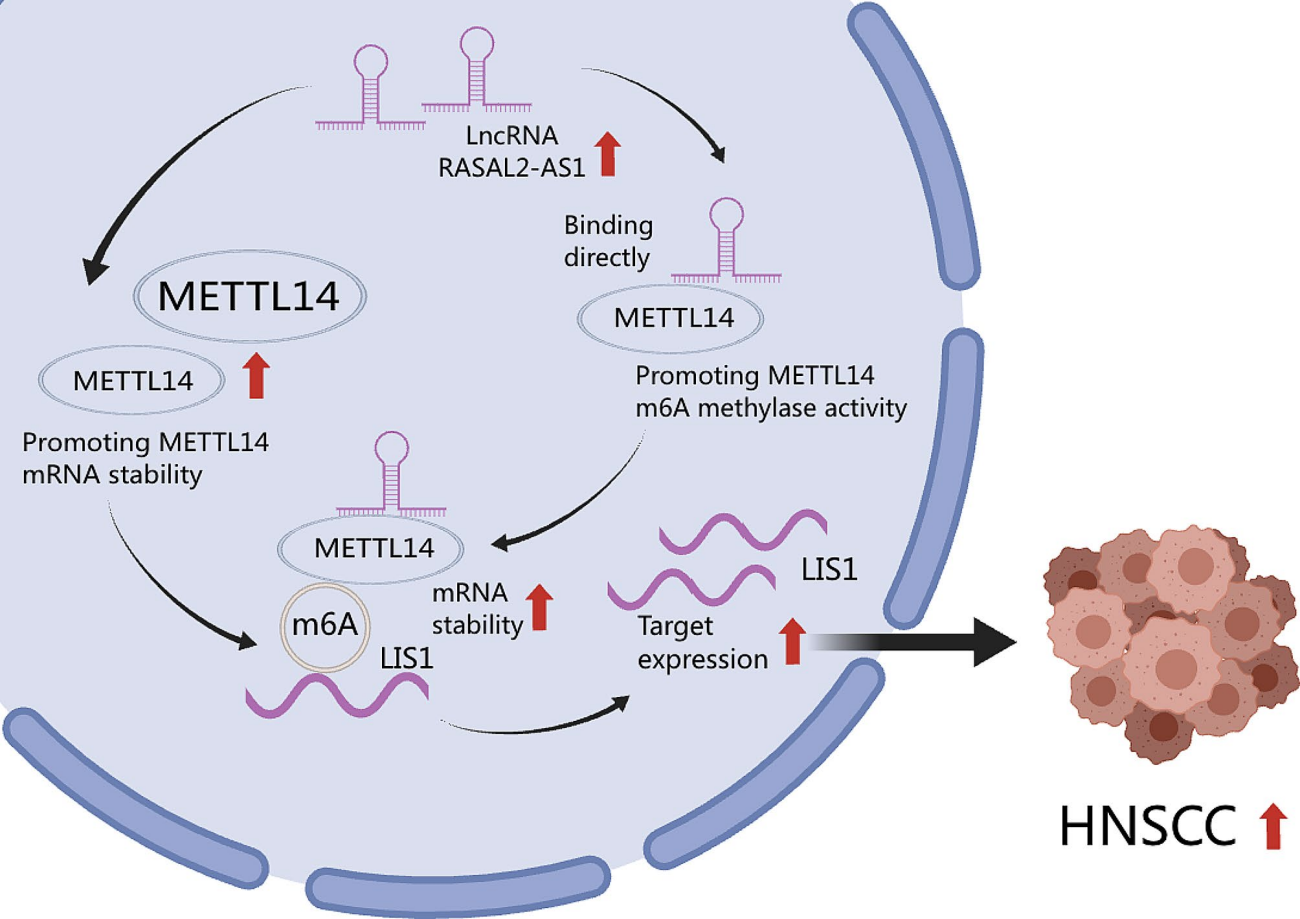
The mechanism diagram of LncRNA RASAL2-AS1 promotes METTL14-mediated m6A methylation in the proliferation and progression of head and neck squamous cell carcinoma

In summary, our findings suggested that METTL14, a central member of the m6A methyltransferase complex, is recruited by RASAL2-AS1 through direct binding, thereby promoting the stability and expression of its downstream target LIS1. (Fig. 7), suggesting that the lncRNA RASAL2-AS1/ METTL14/LIS1 axis might be a potential therapeutic target for head and neck squamous cell carcinoma patients.

## Discussion

HNSCC has a high mortality and morbidity rate. Approximately 60% of patients with HNSCC develop distant metastasis of tumor cells when they reach advanced stages [29, 30]. To date, the underlying mechanism and biological characteristics of lncRNA RASAL2-AS1 in cancers by m6A methylated modification have not been reported yet. In this study, we uncovered that RASAL2-AS1 exerts its ascendant functions as an oncogenic regulator involved in HNSCC tumorigenesis and development.

It has been reported that the most important prognostic factor for patients with HNSCC is the clinical stage of disease (TNM stage). However, survival is variable for patients with the same stage, and more prognostic markers are needed [31, 32]. Consistently, our study confirmed the close relationship between lncRNA RASAL2-AS1 and HNSCC survival. Moreover, RASAL2-AS1 expression was higher in Cal-27 and SCC-25 cells and HNSCC tissues. Long non-coding RNA (lncRNA) plays a vital role in tumor proliferation, migration, and treatment [33]. Given higher tissue specificity and easier detection than mRNA, lncRNA is more suitable as a biomarker for tumor diagnosis and prognosis [34, 35]. In subsequent functional assays, we demonstrated that lncRNA RASAL2-AS1 knockdown could suppress cell proliferation, migration and arrest cell cycle in the G1 phase, while RNA RASAL2-AS1 overexpression could reverse above effects.

M6A methylation stands as the predominant mRNA modification in eukaryotic cells, characterized by a reversible chemical process dynamically governed by the coordinated activity of m6A methyltransferases and demethylases [36]. Notably, m6A “writer” proteins, namely METTL3 and METTL14, have been identified as dysregulated factors in bladder, gastric, and pancreatic cancers [37–41]. Recent studies have increasingly revealed the pivotal regulatory roles of lncRNAs in tumorigenesis and progression. These roles emerge through interactions with m6A regulators via m6A methylation modifications [42, 43]. Interestingly, our results that inhibiting METTL14 expression can effectively counteract the biological effects induced by overexpression of the RASAL2-AS1 gene. For instance, METTL14 has been demonstrated to restrain the growth and metastasis of kidney cancer by reducing the expression of lncRNA NEAT1 [44]. In addition, RASAL2-AS1 has been identified as a methylation-driven gene closely associated with the onset and progression of HCC. A recent study established the m6A methylation group of lncRNA based on tissue samples and designed the expression profile of lncRNA/mRNA to explore the potential function of lncRNA regulated by m6A in participating in GC initiation. It was found that RASAL2-AS1 may play a regulatory role in GC cell proliferation [45, 46].

Previous research has identified LIS1 as a key protein involved in multiple pathways, including its association with molecular motor cytoplasmic dynein, signaling pathways, and the platelet activation pathway. It has recently been discovered that LIS1 can actively promote tumor cell migration, invasion, proliferation, and metastasis. However, its role in HNSCC remains unclear. Corresponding to our consequence, silencing RASAL2-AS1 in Cal-27 and SCC-25 cells resulted in significant LIS1 downregulation, while RASAL2-AS1 overexpression reversed the phenomenon, and these were further corroborated by western blot analysis and GEPIA platform. Regarding lncRNA RASAL2-AS1, is identified as a key factor inducing tongue squamous cell carcinoma, while the potential for it to become a drug for immunotherapy in tongue squamous cell carcinoma remains an open question. This forms a novel direction for our subsequent research.

Detailedly, in Cal27 and SCC-25 cells (tongue squamous carcinoma, TC), RASAL2-AS1 was upregulated accompanied with the elevated mRNA stability and protein expression of methyltransferase-like 4 (METTL14) associated with m6A methylation levels, which led to the promotion of cell proliferation and colony formation, as well as influencing cell G1 phase arrest. Mechanistically, RASAL2-AS1 specifically bound with METTL14 protein. Thereafter, the METTL14, regulated by RASAL2-AS1, promoted the methylation level and the stability of m6A, irritating its target genes such as LIS1.

Such promotion could be almost recovered by exposure on RASAL2-AS1 overexpression and METTL14 knockdown in vitro, subsequently reversing the expression patterns of the above genes. The effects of RASAL2-AS1/METTL14/LIS1 (PAFAH1B1) on the biological characteristics of HNSCC were validated through phenotype experiments including MTT assay, colony formation assay, transwell migration assay, scratch assay, flow cytometry, and others. The interaction among RASAL2-AS1, METTL14, and LIS1 (PAFAH1B1) was confirmed through RIP immunoprecipitation. The results of human immunohistochemistry indicated that there is high expression of METTL14 and LIS1 in tongue cancer tissues compared to normal tissues. In tumor xenograft assays using nude mice, tumor tissue size was measured upon tumor removal, and immunohistochemical analysis indicated that RASAL2-AS1 overexpression promoted HNSCC cell proliferation and growth, while silencing of METTL14 inhibited these effects. These findings strongly suggest that RASAL2-AS1 targets METTL14 regulating LIS1 signaling, thereby has a profound influence on the development of HNSCC.

In this current study, we unveil a novel molecular mechanism governed by the RASAL2-AS1/METTL14/LIS1 signaling pathway axis, which plays a crucial role in the developmental process of HNSCC. METTL14, a central member of the m6A methyltransferase complex, is recruited by RASAL2-AS1 through direct binding, thereby promoting the stability and expression of its downstream target LIS1. Consequently, this cascade leads to cell proliferation, migration, colony formation, and impacts the cell cycle both *in vitro and in vivo*. As a result, our findings propose that lncRNAs and their associated binding proteins orchestrate the regulation of downstream genes through m6A-dependent methylated modifications. This revelation presents a new avenue and strategy for investigating the underlying molecular mechanisms of HNSCC progression and offers potential therapeutic avenues.

### Electronic supplementary material

Below is the link to the electronic supplementary material.

Supplementary Material 1

Supplementary Material 2

## Data Availability

All data and materials can be obtained from the corresponding author.

## Abbreviations

BSA: bovine serum albumin
DMEM: Dulbecco’s modified Eagle’s medium
FBS: fetal bovine serum
GC: gastric cancer
GEPIA: Gene Expression Profiling Interactive Analysis
HCC: hepatocellular carcinoma
HNSCC: Head and neck squamous cell carcinomas
IHC: Immunohistochemistry analysis
LIS1: Lissencephaly 1
LncRNA: Long non-coding RNA
m6A: N6-methyl-adenosine
METTL14: METTL14 methyltransferase-like 14
METTL3: METTL3 methyltransferase-like 3
miRNA: microRNA
mRNA: Messenger RNA
MTT: Methylthiazolyl tetrazolium
OSCC: oral squamous cell carcinoma
PBS: phosphate-buffered saline
PMSF: Phenylmethanesulfonyl fluoride
PVDF: polyvinylidene difuoride
RIP: RNA immunoprecipitation
RIPA: Radio Immunoprecipitation Assay
RT-PCR: Reverse transcription-polymerase chain reaction
SDS-PAGE: sodium dodecyl sulfate-polyacryamide gel electrophoresis
siRNA: Small interfering RNA
TAE Buffer: Tris-acetate-EDTA Buffer
TBST: Tris buffered saline containing Tween
TC: tongue squamous carcinoma
TCGA: The Cancer Genome Atlas
UCSC: University of California Santa Cruz
