# Supplementary material for: LncRNA RASAL2-AS1 promotes METTL14-mediated m6A methylation in the proliferation and progression of head and neck squamous cell carcinoma
Source: Cancer Cell Int. 2024 Mar 25;24:113. doi: 10.1186/s12935-024-03302-8 (PMC10962181; doi:10.1186/s12935-024-03302-8)
Supplement: Supplementary file 1 — Supplementary Material 1 [file 12935_2024_3302_MOESM1_ESM.docx]

**Table 1** **Primer sequences**

| Gene name | Primer sequences (5′-3′) |
| --- | --- |
| RASAL2-AS1 | F: 5′-TCCTAATGCTCTCCCTCCCCTTTC-3′ |
|  | R: 5′-AACGATGAGAACACATGGACACAGG-3′ |
| GADPH | F: 5ʹ-CGACAGTCAGCCGCATCTT-3′ |
|  | R: 5ʹ-CCAATACGACCAAATCCGTTG-3′ |
| METTL14 | F: 5ʹ-GAACACAGAGCTTAAATCCCCA-3′ |
|  | R:5ʹ-TGTCAGCTAAACCTACATCCCTG-3′ |
| METTL3 | F: 5ʹ-CCAGCACAGCTTCAGCAGTTCC-3′ |
|  | R:5ʹ-GCGTGGAGATGGCAAGACAGATG-3′ |
| WTAP | F: 5ʹ-ACTGGCCTAAGAGAGTCTGAAG-3′ |
|  | R:5ʹ-GTTGCTAGTCGCATTACAAGGA-3′ |
| LIS1 | F:5ʹ-GGGAAGTTTATTTTGAGTTGTGCTG-3′ |
|  | R:5ʹ-AGGGTGCCGTCTTGTGGAA-3′ |

**Table S2 Relationship between lncRNA RASAL2-AS1 expression and clinical features in patients with head and neck cancer**

Characteristics LncRNA RASAL2-AS1 expression Total(N=488) pvalue

high(N=244) low(N=244)

Age at initial pathologic diagnosis

Mean±SD 60.00±11.93 61.95±11.90 60.97±11.94

Median[min-max] 60.00[19.00,88.00] 61.00[28.00,90.00] 61.00[19.00,90.00]

gender.demographic 0.92

female 67(13.73%) 65(13.32%) 132(27.05%)

male 177(36.27%) 179(36.68%) 356(72.95%)

clinical_M 0.86

M0 236(48.36%) 236(48.36%) 472(96.72%)

M1 3(0.61%) 2(0.41%) 5(1.02%)

MX 5(1.02%) 6(1.23%) 11(2.25%)

clinical_N 0.006*

N0 114(23.36%) 125(25.61%) 239(48.98%)

N1 44(9.02%) 36(7.38%) 80(16.39%)

N2 17(3.48%) 2(0.41%) 19(3.89%)

N2a 6(1.23%) 12(2.46%) 18(3.69%)

N2b 38(7.79%) 38(7.79%) 76(15.57%)

N2c 20(4.10%) 21(4.30%) 41(8.40%)

N3 4(0.82%) 3(0.61%) 7(1.43%)

NX 1(0.20%) 7(1.43%) 8(1.64%)

clinical_T 0.41

T1 13(2.66%) 20(4.10%) 33(6.76%)

T2 64(13.11%) 80(16.39%) 144(29.51%)

T3 70(14.34%) 61(12.50%) 131(26.84%)

T4 14(2.87%) 11(2.25%) 25(5.12%)

T4a 80(16.39%) 71(14.55%) 151(30.94%)

T4b 2(0.41%) 1(0.20%) 3(0.61%)

TX 1(0.20%) 0(0.0e+0%) 1(0.20%)

Clinical stage 0.35

Stage I 6(1.23%) 13(2.66%) 19(3.89%)

Stage II 43(8.81%) 52(10.66%) 95(19.47%)

Stage III 54(11.07%) 48(9.84%) 102(20.90%)

Stage IVA 131(26.84%) 126(25.82%) 257(52.66%)

Stage IVB 6(1.23%) 3(0.61%) 9(1.84%)

Stage IVC 4(0.82%) 2(0.41%) 6(1.23%)

Neoplasm histologic grade 0.2

G1 20(4.12%) 35(7.22%) 55(11.34%)

G2 151(31.13%) 144(29.69%) 295(60.82%)

G3 65(13.40%) 52(10.72%) 117(24.12%)

G4 1(0.21%) 1(0.21%) 2(0.41%)

GX 7(1.44%) 9(1.86%) 16(3.30%)

Vital status.demographic 0.01*

Alive 121(24.80%) 150(30.74%) 271(55.53%)

Dead 123(25.20%) 94(19.26%) 217(44.47%)

**P<0.05*
