# Supplementary figures and images for: LncRNA RASAL2-AS1 promotes METTL14-mediated m6A methylation in the proliferation and progression of head and neck squamous cell carcinoma
Source: Cancer Cell Int. 2024 Mar 25;24:113. doi: 10.1186/s12935-024-03302-8 (PMC10962181; doi:10.1186/s12935-024-03302-8)

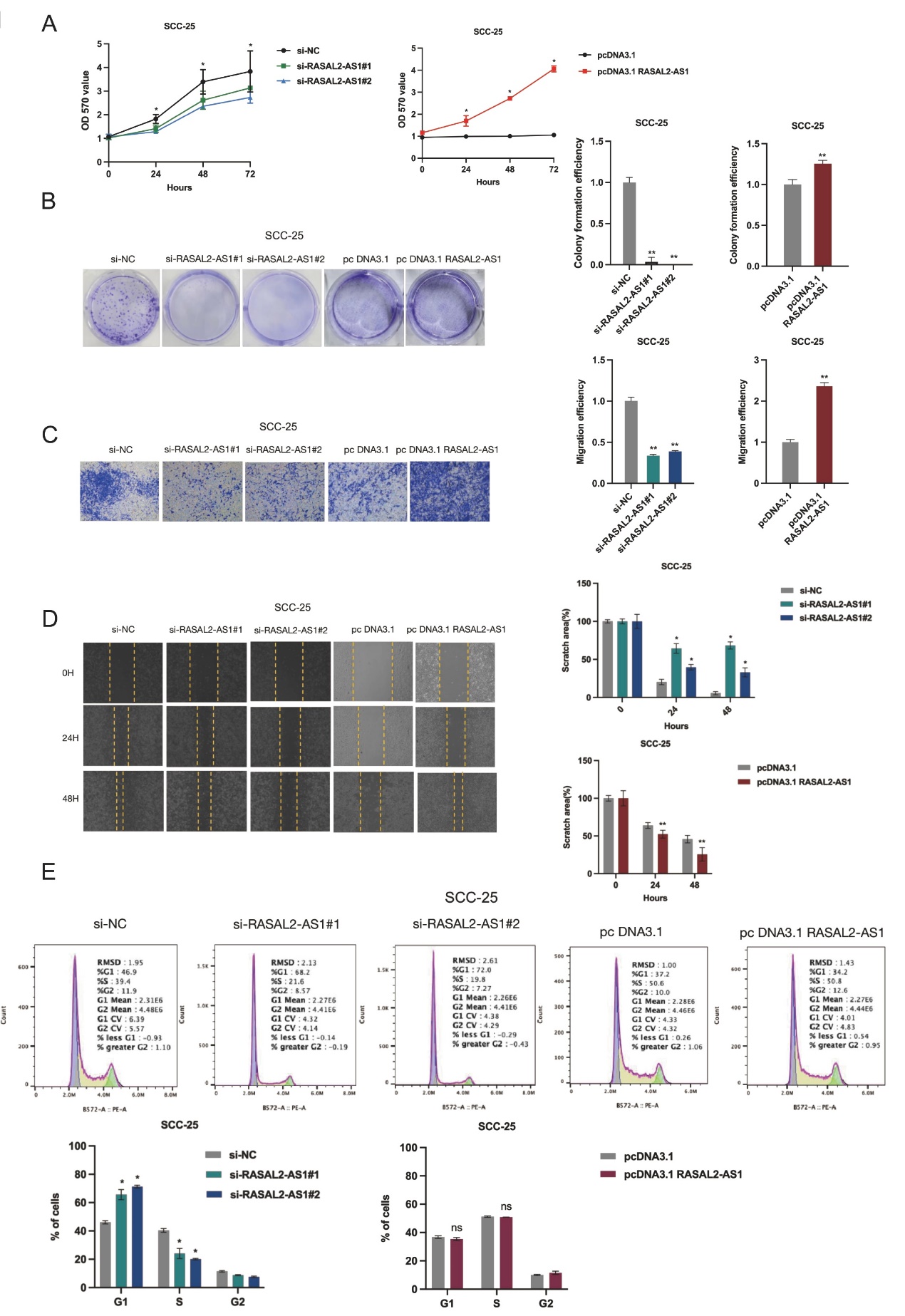


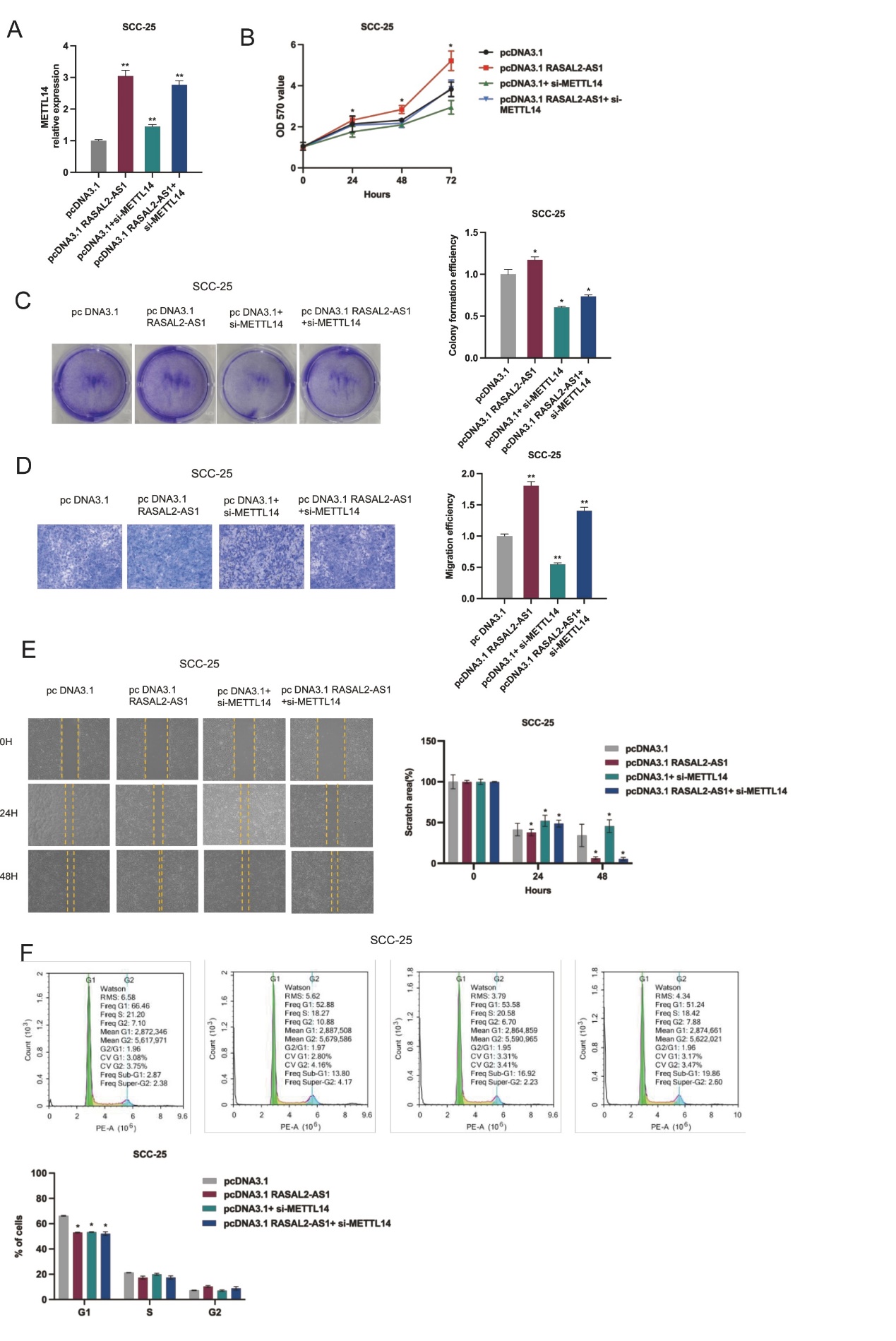

Supplement: Supplementary file 2 — Supplementary Material 2 [file 12935_2024_3302_MOESM2_ESM.docx]
